# Modelling the potential impact of a sugar-sweetened beverage tax on stroke mortality, costs and health-adjusted life years in South Africa

**DOI:** 10.1186/s12889-016-3085-y

**Published:** 2016-05-31

**Authors:** Mercy Manyema, Lennert J. Veerman, Aviva Tugendhaft, Demetre Labadarios, Karen J. Hofman

**Affiliations:** PRICELESS-SA, MRC/Wits Rural Public, Health and Health Transitions Research Unit, School of Public Health, Faculty of Health Sciences, University of the Witwatersrand, Johannesburg, South Africa; School of Public Health, University of Queensland, Brisbane, Queensland Australia; Population Health, Health Systems and Innovation (PHHSI), Human Sciences Research Council, Cape Town, South Africa

**Keywords:** Sugar-sweetened beverages, Stroke, Modelling, Mortality, Health care costs

## Abstract

**Background:**

Stroke poses a growing human and economic burden in South Africa. Excess sugar consumption, especially from sugar-sweetened beverages (SSBs), has been associated with increased obesity and stroke risk. Research shows that price increases for SSBs can influence consumption and modelling evidence suggests that taxing SSBs has the potential to reduce obesity and related diseases. This study estimates the potential impact of an SSB tax on stroke-related mortality, costs and health-adjusted life years in South Africa.

**Methods:**

A proportional multi-state life table-based model was constructed in Microsoft Excel (2010). We used consumption data from the 2012 South African National Health and Nutrition Examination Survey, previously published own and cross price elasticities of SSBs and energy balance equations to estimate changes in daily energy intake and BMI arising from increased SSB prices. Stroke relative risk, and prevalent years lived with disability estimates from the Global Burden of Disease Study and modelled disease epidemiology estimates from a previous study, were used to estimate the effect of the BMI changes on the burden of stroke.

**Results:**

Our model predicts that an SSB tax may avert approximately 72 000 deaths, 550 000 stroke-related health-adjusted life years and over ZAR5 billion, (USD400 million) in health care costs over 20 years (USD296-576 million). Over 20 years, the number of incident stroke cases may be reduced by approximately 85 000 and prevalent cases by about 13 000.

**Conclusions:**

Fiscal policy has the potential, as part of a multi-faceted approach, to mitigate the growing burden of stroke in South Africa and contribute to the achievement of the target set by the Department of Health to reduce relative premature mortality (less than 60 years) from non-communicable diseases by the year 2020.

**Electronic supplementary material:**

The online version of this article (doi:10.1186/s12889-016-3085-y) contains supplementary material, which is available to authorized users.

## Background

Stroke is a major cause of disability and death worldwide. The Global Burden of Disease Study (GBD) shows that approximately 11.6 million cases of ischaemic stroke (65 % in low-to-middle income countries, LMICs) and 5.3 million of haemorrhagic stroke (80 % in LMICs) occurred worldwide in 2010. Sixty-four percent of the disability-adjusted life years (DALYs) due to ischaemic stroke and 86 % of DALYs due to haemorrhagic stroke were lost in LMICs [1]. In sub-Saharan Africa (SSA), more than 30 % of stroke patients die within the first month, less than 60 % of patients are alive after six months and by one year less than 50 % are still alive [2]. This global burden is projected to increase to 23 million first-ever strokes and 7.8 million deaths by 2030 [3].

The burden is also increasing in South Africa. In 2000, stroke (mostly haemorrhagic) was the third leading cause of death after HIV/AIDS and ischemic heart disease [4]. In 2008, a modelling study showed that 75 000 new cases of stroke occurred in that year, with a third of these being fatal within 28 days. Of the 350 000 stroke survivors, 35 % had moderate to severe disability due to the condition [4]. An estimated 33 500 strokes occurred in rural South Africa in 2011 [5].

Stroke poses a significant human and economic burden. The total direct and indirect cost of stroke for 2008 in the United States of America (USA) was estimated at USD65.5 billion, and 27 billion Euros in 27 European Union countries [6]. The estimated cost of care for stroke in SSA is USD157 per episode [2]. Direct costs include the cost of physicians and other health professionals, acute and long-term care, medications and other medical durables. Additional indirect costs include lost productivity resulting from morbidity and mortality and the costs of informal care by families and communities. Affecting mostly the economically productive age group especially in LMICs, stroke leaves about 65 % of its victims disabled leading to increased loss of manpower both at individual, household and societal levels [7] which adversely affects productivity and income, and hampers development. It also affects social relationships and economic status.

Hypertension is the most prevalent, independent and modifiable risk factor for stroke at the population level in SSA [3, 8] with over 50 % of stroke cases in South Africa attributable to hypertension [9]. Other risk factors for stroke include diabetes, smoking, dyslipidaemia, obesity and heavy alcohol consumption [2]. Increasing evidence however shows a significant link between excess sugar consumption, especially from sugar-sweetened beverages (SSBs), and risk of cardiovascular disease (CVD), including stroke. Data from the GBD estimates show that in 2010 approximately 184 000 deaths worldwide were attributable to SSB consumption, with almost 25 % of these due to CVD [10]. Further longitudinal evidence suggests a positive association between SSB consumption and increased stroke risk and mortality [11–13]. The relationship between SSB consumption and stroke may be mediated through weight gain and/or hypertension [14–18]. However, an independent effect may arise from the large amounts of highly absorbable sugars found in SSBs which contribute to high glycaemic load and may lead to inflammation and cardiovascular changes [16, 19, 20].

Globally, consumption of SSBs has increased alongside the increase in non-communicable disease (NCD) prevalence. Between 2005 and 2010, added sugar and sucrose-sweetened beverage consumption increased in both urban and rural areas in South Africa, with a corresponding increase in NCD risk factors [21]. The proportion of adults drinking SSBs in rural areas doubled from 2005–2010. Consumption of Coca-Cola products in South Africa increased from 183 per person per year in 2002 to 260 products in 2012 putting South Africa in the top ten consumers of Coca-Cola products [22]. These estimates are based on a USA eight fluid ounce serving or 250 ml. SA Euromonitor data also show a 16 % increase in soft-drink off-trade sales from 3,620 million to 4,206 million litres between 2008 and 2013 respectively [23].

A tax on SSBs is currently being advocated by policy makers and public health experts world-wide as an effective tool to reduce obesity. The South African National Department of Health (DOH), has included this as a cost effective policy intervention as part of its Strategic Plan for the Prevention and Control of NCDs, 2013–2017 [24]. Research shows that price changes due to taxation or subsidies can modify consumption and potentially lead to positive diet and weight outcomes [25, 26]. In addition, modelling evidence suggests that taxing SSBs has the potential to reduce obesity [27–30].

Mexico introduced a tax on SSBs in January 2014 and observational results show an average reduction of 6 % in the purchase of taxed SSBs during 2014 [31]. This reduction accelerated over the course of the year to reach 12 % by December 2014. Household SSB purchases decreased across all socioeconomic levels although the greatest decrease was among the lowest socioeconomic group which achieved a reduction of 17 % by the end of the year [31]. The amount of the tax was one peso ($.07 USD) per liter, roughly equivalent to a 10 % increase in price.

As a leader in promulgating tobacco taxes, the implementation of taxes on tobacco in South Africa has resulted in an aggregate decrease in cigarette consumption of 41 % and a per capita decrease of 66 % over two decades from 1990 [32]. This evidence suggests that fiscal levers, as part of a multi-pronged approach, can influence consumption. The aim of this study was to estimate the impact of an SSB tax on the burden of stroke in South Africa through the reduction of SSB consumption and reduction of population mean body mass index (BMI).

## Methods

An SSB was defined as a non-alcoholic drink with added sugar. This comprised carbonated sweetened drinks, sweetened fruit juices and squash concentrates. The analysis method involved two steps. The first step was to quantify the impact of a 20 % SSB tax on the population BMI distribution through changes in consumption and energy intake. The second step was estimating the changes in stroke outcomes due to the tax-induced changes in BMI distribution using a life table-based Markov model implemented in Microsoft Excel (2010). In the model, a reference population with the BMI distribution and disease pattern of the South African adult population aged 15 years and older in 2012 was compared with an identical intervention population which received the 20 % tax intervention.

**Fig. 1:**
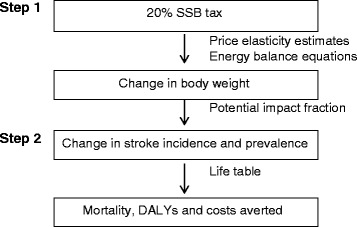
Analytical framework of the model. Step 1, estimation of the change in consumption and change in body weight resulting from the SSB tax; Step 2, estimation of the changes in stroke incidence, prevalence, mortality, costs saved and healthy years of life gained

Figure 1 is the schematic of the model showing the modelling steps for the intervention population. The reference population is modelled similarly except that no changes in BMI are incorporated since no trend in BMI was applied. The difference in health outcomes between the two populations is attributed to the tax.

### Intervention

A tax rate of 20 % was modelled for a period of 20 years, assuming a 100 % pass on rate. Tax rates of 10 and 20 % have been modelled in the past, with pass on rates ranging between 80 and 100 % [27–29].

### Change in SSB consumption

Data from the 2012 South African National Health and Nutrition Examination Survey (SANHANES-1), a baseline cross-sectional survey of the SANHANES series, were used to derive baseline consumption of SSBs, milk and unsweetened fruit juice in adults aged 15 years and older [33]. The procedures for data collection and for extracting data for the model have been described elsewhere [30]. Changes in consumption of SSB, milk, diet drink and unsweetened fruit juice resulting from an increased SSB price were estimated using own and cross price elasticities previously published in a systematic review and meta-analysis [26]. Price elasticity estimates from South Africa were not available. Cross price elasticities were used to estimate the replacement of SSBs with other drinks by consumers given an SSB price increase. The price elasticity estimate values and standard deviations are shown in Additional file 1: Table S1.

### Change in energy intake

Average energy density estimates for each drink category in kilojoules were used to translate the change in SSB consumption into change in energy intake. The energy density of SSBs was assumed to be 1800 kilojoules (kJ)/litre, 1340 kJ/l for unsweetened juice (both based on calculations using several Coca-Cola products) and 2540 kJ/l for whole milk based on values given by Parmalat South Africa [34]. Coca-Cola accounts for approximately 60 % of all off-trade soft drink sales in South Africa [35]. In addition, personal communicator, Dr Celeste Naude, Centre for Evidence-based Health, Stellenbosch University, calculated the mean energy density for an SSB to be 188 kJ per 100 ml (SD 40) using energy density values of a sample of 90 carbonated drinks, sports drinks, concentrates, iced teas and sweetened fruit juices, obtained from the South African Medical Research Council Food Data System [36] and nutrition information provided on beverage labels. Based on an analysis of dietary surveys in South Africa which shows that more full cream milk is consumed per capita than skim milk, we assumed that all milk consumed was full cream milk [37]. The changes in energy intake for each beverage type were summed up to give the net change in energy intake.

### Change in energy balance and BMI

A study by Swinburn et al. shows that a daily change in energy intake of 94 kJ/day (SD 2.96) is associated with a change in body weight of 1 kg in equilibrium for adults [38]. We used this estimate to calculate the changes in body weight resulting from the changes in energy intake. Our model assumes that the population will lose weight until a new equilibrium in line with lower energy intake is reached [38]. Baseline BMI data for adults aged 15 years and above were extracted from the 2012 Wave 3 National Income Dynamics Study (NIDS) [39] and fitted to the log-normal distribution using previously published procedures, the log-normal distribution being selected above the gamma distribution for better fitting properties [30]. We used data from previous waves of the NIDS (2008 and 2010) to estimate the change in the standard deviation of the mean (BMI) as a function of mean BMI to predict shifts in the BMI distribution of the population arising from the intervention, previously published, [30].

### Change in stroke incidence, prevalence and mortality

The potential impact fraction (PIF), defined as the proportional change in disease risk due to change in exposure to a related risk factor, was used to estimate the change in stroke incidence resulting from shifts in BMI [40]. The Excel add-in JanB [41], which calculates population risk by integrating the product of a continuous risk factor prevalence distribution and a relative risk function was used to calculate the PIF based on the age and sex-specific changes in BMI distributions due to the 20 % tax. We used relative risks of stroke from the GBD 2010 study [42]. The PIF estimates were then used to calculate the changes in stroke incidence. Corresponding changes in prevalence and mortality were calculated in the life table model. Previously published estimates of baseline stroke incidence, prevalence and case fatality rate for SA were used [4]. Case fatality rate is defined as the annual rate at which prevalent cases died. We also obtained from the same study estimates of the proportion of stroke cases that die within 28 days and incorporated these into the model. The disease relative risk, incidence, prevalence and case fatality rate estimates used in the model are presented in Additional file 1: Table S2.

### Health-adjusted life years and health care costs

The changes in stroke prevalence and mortality in the intervention population influence total mortality rates and the average health-related quality of life at each age and sex, and therefore the total number of disability-adjusted life years lived by the cohort. In the life table, the populations are divided into 5-year cohorts and each cohort is simulated until death or 100 years of age. Adjustments for time spent in poor health due to disease or injury are made at each age based on prevalent life years lived with disability (pYLD) derived from the 2010 GBD data for South Africa [43]. The health-adjusted years of life gained due to the intervention are the difference in health-adjusted years of life lived between the reference and intervention populations. Population estimates by age and sex for 2012 were obtained from Statistics South Africa. An average disability weight for stroke of 0.28 was obtained from a previous study [4]. Unpublished all-cause mortality rates were obtained from the SA Medical Research Council (MRC).

Using a health sector perspective, we estimated baseline stroke health care costs using data from the South African Government Employees Medical Scheme (GEMS). We obtained data showing the average amount in South African rands (ZAR) claimed for stroke per member in 2012 (unpublished data). We assumed the GEMS costs to be private sector costs based on the fact that over 90 % of medical aid members use private sector facilities. Under the assumptions that 18 and 82 % of the South African population uses private and public sector facilities respectively [44] and, according to expert opinion, that public sector costs are approximately 70 % of private sector costs, we calculated weighted average costs of stroke in South Africa. Neither the health care costs nor the DALYs were discounted. The pYLD, health care costs and mortality estimates used in the model are given in Additional file 1: Table S3.

### Uncertainty and sensitivity analysis

We estimated ninety-five percent uncertainty intervals (UI) using Monte Carlo simulation using the Ersatz programme (Barendregt JJ, Brisbane 2007), varying the own and cross price elasticity estimates, the conversion factor between energy consumption change and weight change, the consumption estimates by age and sex for all four beverages, and the relative risk estimates.

We performed deterministic sensitivity analysis to assess the effect on the results of varying the tax rate (10 %, 30 %), pass on rate, health care costs (10–20 % increase and decrease), the pYLD estimates, BMI trend estimates, SSB portion size and the discounting rate. We also tested the effect on the results of using the confidence interval lower bound price elasticity estimate.

## Results

### Change in consumption, energy intake and BMI

The average baseline consumption of SSBs in 2012 was 184 ml per day [30]. The change in energy intake and BMI resulting from the tax have been reported previously [30]. The average change in energy was 36 kJ per person per day. Shifts in BMI were slightly greater in women than men on average.

### Change in the burden of stroke

Our model estimates that without the tax intervention, there would be approximately 6 400 000 new adult stroke cases over 20 years, compared to 6 300 000 cases with the intervention. This translates to relative changes of between 1.0 and 1.8 % (Table 1). The number of new cases and relative changes would be higher in females than males.

**Table 1:** Percentage reduction in incident and prevalent stroke cases and mortality

Year	Incidence	Incidence	Prevalence	Prevalence	Mortality	Mortality
	Male (%)	Female (%)	Male (%)	Female (%)	Male (%)	Female (%)
1	1.14	1.80	0.25	0.39	0.77	1.24
	(0.73–1.64)	(1.20-2.50)	(0.16–0.35)	(0.26–0.53)	(0.49–1.11)	(0.82-1.73)
5	1.09	1.70	0.89	1.33	1.02	1.66
	(0.69–1.65)	1.00–2.39)	(0.59–1.30)	(0.87–1.87)	(0.65–1.52)	(0.99–1.73)
10	1.06	1.67	1.02	1.50	1.08	1.74
	(0.67–1.54)	(1.07–2.52)	(0.68–1.54)	(0.99–2.10)	(0.71–1.60)	(1.11–2.51)
15	1.01	1.63	1.04	1.53	1.07	1.73
	(0.64–1.33)	(0.99–2.32)	(0.70–1.50)	(1.03–2.22)	(0.71–1.60)	(1.07–2.51)
20	1.01	1.67	1.03	1.55	1.07	1.76
	(0.63–1.42)	1.02–2.37)	(0.68–1.42)	(0.96–2.14)	(0.71–1.46)	(1.07–2.46)

**Fig. 2:**
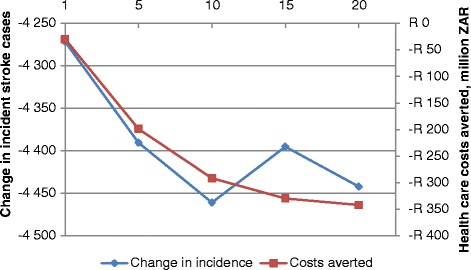
Stroke-related health care costs averted and change in incident stroke cases over time (20 years). Left vertical axis, change in stroke incident cases; right vertical axis, health care costs averted

The health care costs that may potentially be saved are substantial, with more gains being made early on. Figure 2 presents the change in incidence and stroke-related health care costs averted.

In year 20 of the intervention, a potential ZAR342 million (USD27 million) (95 % UI: ZAR222–463 million, USD 18–37 million) may be saved, compared to ZAR30 million (USD2 million) (95 % UI: ZAR21–ZAR39 million, USD1.7–3.1 million) in year one, for a total of over ZAR5 billion (USD 400 million) over the entire 20 year period (95 % UI: ZAR3.7–7.2 billion, USD296–576 million).

**Fig. 3:**
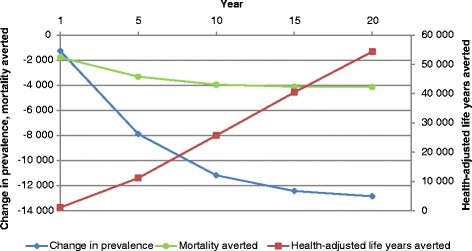
Change in prevalent stroke cases and annual deaths and health-adjusted life years averted over time (20 years). Left vertical axis, change in prevalence and mortality averted over time; right vertical axis, health-adjusted life years averted

The tax is predicted to reduce the number of prevalent stroke cases in adults by approximately 13 000 (95 % UI: 8 400–17 000) by year 20. We estimate that over 72 000 deaths may be averted by the tax over the same period (95 % UI: 51 000–98 000). Figure 3 shows the deaths and DALYs averted compared to changes in prevalence over time.

The annual number of deaths stabilises over the 20 year period. The change in prevalent cases also shows a similar trend. However, the gains in healthy years of life consistently increase. The model estimates that about 550 000 stroke-related DALYs will be averted over a 20 year period (95 % UI: 361 000–767 000).

### Sensitivity analysis

The results of the sensitivity analyses are shown in Table 2.

**Table 2:** Sensitivity analysis of the effect on the outcome of variation in model parameters

Parameter tested	Total stroke incidence over 20 years	Total stroke DALYs over 20 years	Total stroke prevalence over 20 years	Total stroke health care costs over 20 years (billion ZAR)
Tax rate				
10 %	−50 457	321 750	−7 538	−3.0
20 %	−85 766	546 762	−12 860	−5.1
30 %	−109 435	706 570	−16 168	−6.6
Discount rate				
0 %	−85 766	546 762	−12 860	−5.1
1 %	−87 824	494 539	−13 165	−4.8
2 %	−88 646	441 074	−13 197	−4.3
3 %	−89 648	397 838	−13 300	−4.0
SSB portion size				
200 ml	−19 395	129 488	−2 760	−1.0
250 ml	−46 150	296 910	−6 882	−2.7
330 ml	−85 766	546 762	−12 860	−5.1
500 ml	−174 008	1 107 665	−26 115	−10.6
Health care costs				
120 %	−85 766	546 762	−12 860	−6.3
110 %	−85 766	546 762	−12 860	−6.0
100 %	−85 766	546 762	−12 860	−5.1
90 %	−85 766	546 762	−12 860	−4.8
80 %	−85 766	546 762	−12 860	−4.1
pYLD				
100 %	−85 766	546 762	−12 860	−5.1
105 %	−85 766	556 926	−12 860	−5.1
110 %	−85 766	541 548	−12 860	−5.1
BMI trend				
0	−85 766	546 762	−12 860	−5.1
0.1	−99 427	622 762	−15 432	−5.8
0.2	−109 060	673 237	−17 269	−6.1
0.3	−113 568	707 078	−18 015	−6.3
Pass on rate				
80 %	−74 637	477 949	−11 146	−4.5
90 %	−81 846	524 691	−12 198	−4.9
100 %	−85 766	546 762	−12 860	−5.1
Lower own price elasticity (−0.85)	−32 205	211 543	−4 686	−1.8

A higher tax rate would result in higher gains with diminishing returns across all parameters and a higher discount rate would generate the opposite effect, as future health gains are valued less. The effect of changing the pYLD on the results is negligible. Incorporating an upward BMI trend in the model would also lead to greater gains. However, if manufacturers and retailers do not pass on the full amount of the tax, the gains would be less. Similarly, if purchasing behaviour is not strongly influenced by the price increase (lower price elasticity) then the effects of the tax would be lower.

## Discussion

Our model predicts that an SSB tax may avert approximately 550 000 stroke-related DALYs, 72 000 deaths and over ZAR5 billion, (USD400 million) in health care costs over 20 years (USD296–576 million). The number of incident stroke cases may be reduced by approximately 85 000 and prevalent cases by about 13 000 over 20 years.

The impact of taxing SSBs has been assessed for obesity and diabetes but this is the first study to assess the impact of an SSB tax on the burden of stroke. With the announcement by the South African government to introduce a 20 % SSB tax in April 2017 [45], this body of work will be of use to the policy makers.

Taxing SSBs was predicted to reduce the number of obese adults by over 220 000 in South Africa (20 % tax) [30], 9 900 in Ireland (10 % tax) [29] and 180 000 in the United Kingdom (20 % tax) [28]. A 20 % tax has been projected to potentially reduce the prevalence of diabetes by 4.0 % in South Africa and by 1.6 % in India [27, 46]. Our results show decreases in stroke prevalence of between 0.32–1.31 % and compare well with the other studies despite different impact sizes. Several reasons may explain this difference in impact size of an SSB tax on diabetes versus stroke. Firstly, the relative risk of diabetes given increasing BMI is higher than for stroke. Secondly, the comparisons are between different countries and differing BMI distributions and baseline prevalence rates of obesity play a role. It may be due to the use of different methods in the studies and lastly, stroke has a high 28 day mortality rate (accounted for in our model) therefore only those who survive the first 28 days can continue to benefit from the tax.

### Study strengths and limitations

The strengths of our study include the use of South African data for baseline consumption and BMI and stroke health care costs, the use of price elasticities to account for substitution to other drinks and modelling the effect of the tax by sex over time. The pYLD estimates from the GBD were also specific to South Africa. We accounted for stroke cases that die within 28 days using previously published data and therefore accounted for these in the DALYs and mortality averted.

Our epidemiological and cost estimate inputs were based on observational data which is subject to information and selection bias. Measures were, however, taken in the nationwide surveys from which we derived BMI and consumption estimates. We also included these in the Monte Carlo simulations or sensitivity analyses.

The price elasticity and relative risk estimates were not specific to South Africa and we included these in the Monte Carlo simulation for uncertainty. Previous studies in the USA and Europe have also used similar price elasticity estimates [25, 28, 29]. In India, a lower estimate was used [27] and using a similar estimate would have led to smaller changes in SSB consumption and reduced changes in the obesity and stroke burden. Briggs et al. [28] found that increasing the price of SSBs would increase and not decrease the demand for diet drinks as assumed in our model. However, since diet drinks are designed to have low caloric content this would have minimal impact on the model. Both the Indian and British studies quoted above used smaller fruit juice cross-price elasticities than our study.

We used proxy estimates for the health care costs of stroke due to the unavailability of data and the effect of variation in these data was tested in sensitivity analyses. We did not account for costs that may result from other diseases or senior care costs in the people who avoid death from stroke due to the intervention. Also not included in the analysis are potential costs of taxation. Accounting for these may attenuate the gains in health care costs.

The baseline consumption data were self-reported which may have led to over- or underestimation of consumption levels as shown by the sensitivity analysis results for SSB portion size. It is possible that we underestimated the baseline disease parameters due to the general paucity of data on stroke in South Africa [4, 5]. We did not model the direct impact of SSB consumption on stroke nor the potential impact of the tax through the mediation of other diseases like type 2 diabetes mellitus (T2DM) and heart disease and other risk factors such as hypertension, which would add considerably to the full impact of the tax given the increasing burden of these conditions in South Africa. The CMS reports an 85 % overall increase prevalence of T2DM between 2006 and 2011 in medical aid beneficiaries and evidence also shows that the burden of hypertension is increasing [4, 5, 44].

Due to unavailability of data, the model did not account for substitution to other possible drink categories such as coffee and tea, flavoured, sweetened milk or to other sweetened foods. This may have potentially led to under or overestimation of the impact of the tax, depending on the size of the shift to the alternative drinks. Other studies show that substitution to non-drink foods does not significantly affect the results [47].

We assumed that the price elasticities would have the same effect across different income groups. Evidence from other studies suggests that the effect may be the same, or demand may be reduced to a greater extent in the lower income groups, while other studies found lower demand only for particular categories of SSBs [27–29, 47].

Some research suggests that increased BMI is associated with a decreased risk of mortality in stroke survivors, the so-called “obesity paradox” [48, 49]. Not much evidence is available on the mechanisms involved but two plausible explanations for the obesity paradox may be collider stratification bias when there is conditioning on disease state in the analysis as well as reverse causality bias [50, 51]. Accounting for the obesity paradox in our model would have attenuated the impact of the tax.

### Study implications

Stroke causes a high mortality and disability burden with about 33 % of cases in South Africa dying within the first 28 days, and more than 75 % dying within 4 years post-event (SSA) [2, 4]. Between 50–65 % of survivors have some form of physical or cognitive disability [6]. Stroke is increasingly affecting those aged 60 years and younger [7]. This leads to loss of work force and poor economic outcomes with the loss of breadwinners, while on the other hand, the prognosis of stroke is worsened by poor economic conditions. Many patients present late and there is poor access to rehabilitation services [2]. This situation demonstrates the need to implement and leverage population-wide interventions such as fiscal or legislature measures to prevent stroke and other NCDs [52].

Although the tax has the potential to be financially regressive, it may potentially be more beneficial to lower income groups in South Africa because of limited access to quality health care. To the extent that low-income individuals are more price sensitive, they will be more likely to reduce their intake of SSBs, and thus experience greater health gain [26]. In Mexico reductions in purchases of taxed SSBs were highest among households of low socio-economic status [31].

An SSB tax would contribute to the multi-pronged approach on NCD prevention envisaged by the DOH [24]. Mandatory salt regulations were passed in South Africa in 2013 and will take effect in 2016 [53]. These regulations have been projected to prevent approximately 7 400 CVD deaths and 4 300 non-fatal strokes annually, amounting to an annual cost saving of ZAR300 million (USD 40 million) [54]. The modelled SSB tax would be complementary to these regulations and result in greater reduction of disability and death due to NCDs and greater cost savings.

The tax has the potential to reduce the burden on the health system. In South Africa, stroke accounts for 5 % of all admissions and 10 % of bed occupancy in adult medical wards [2]. In 2011, the annual cost of treating stroke (excluding rehabilitation) was estimated at ZAR13–16 billion (USD1.0–1.3 million) annually amounting to ZAR16–20 billion in 2015 (USD1.3–1.6 million) [4]. With a total health budget allocation of ZAR157.3 billion in 2015 in South Africa [55], the cost of treating stroke would consume approximately 10–13 % of the budget. Our results estimate a saving of approximately ZAR5 billion over 20 years. Together with other interventions, this would make a significant difference to the health system.

The tax could potentially raise substantial revenue. Preliminary calculations done at PRICELESS-SA, Johannesburg, South Africa, indicate that approximately ZAR7 billion may potentially be raised every year from an SSB tax (unpublished data). Although currently in South Africa tax revenue is not earmarked [56], it can contribute to the national health budget.

The informal and indirect costs of disease are often overlooked. Stroke survivors depend on family and community for support for activities of daily living (ADL) which in turn impacts on their quality of life [6]. Our model did not incorporate these costs and so potentially underestimated the resources that may be saved by the tax. Reducing stroke frequency by preventive measures, as well as reducing stroke mortality and long-term disability by evidence-based acute and post-discharge treatments, is essential to avoid the trend of increase in the stroke burden [6].

An increased SSB price conveys a message that SSBs are unhealthy and are therefore being taxed. This public health message would also contribute to the creation of enabling environment for making better dietary choices.

## Conclusions

Fiscal policy has the potential to mitigate the growing burden of stroke in South Africa and contribute to the achievement of one of the targets set by the South African government to reduce relative premature mortality (less than 60 years) from NCDs by 2020 [24]. The South African government announced plans in February 2016 to introduce a 20 % SSB tax in April 2017 [45]. If implemented as part of a multi-faceted strategy, this far-sighted approach may have a direct impact on obesity and on reducing associated NCDs such as stroke.

### Ethics and consent

This study was a secondary analysis of human participant data collected through two national surveys, the SANHANES-1 for baseline consumption data and NIDS for baseline prevalence of obesity. Both sets of data were anonymised and did not contain any identifying information. The two national surveys themselves independently obtained consent and ethics approval before they commenced [33, 57]. Our manuscript does not contain any individual person’s data.

### Availability of data materials

The NIDS dataset is available from the public access portal of Data First, a unit based at the University of Cape Town, South Africa https://www.datafirst.uct.ac.za/dataportal/index.php/catalog/453. The SANHANES dataset is only available upon request from the Human Sciences Research Council, South Africa, through the following contact: Lucia Lötter, Manager Data Curation, Research Methodology and Data Centre, HSRC; llotter@hsrc.ac.za.

## Additional file

Additional file 1: Estimates of the parameters used in the model.docx. Tables displaying parameter estimates used as inputs for the model. (DOCX 24 kb)

## Abbreviations

BMI: body mass index
CMS: Council for Medical Schemes
CVD: cardiovascular disease
DALY: disability adjusted life years
DOH: National Department of Health
GBD: Global burden of disease
GEMS: Government Medical Aid Scheme
LMIC: low to middle income country
NCD: non-communicable disease
NIDS: National Income Dynamics Study
PIF: potential impact fraction
PRICELESS-SA: Priority Cost Effectiveness Lessons for Systems Strengthening South Africa
pYLD: prevalent years lived with disability
SANHANES: South African National Health and Nutrition Examination Survey
SSA: sub-Saharan Africa
SSB: sugar-sweetened beverage(s)
USA: United States of America
USD: United Sates dollar
ZAR: South African rand
