# Supplementary material for: Modelling the potential impact of a sugar-sweetened beverage tax on stroke mortality, costs and health-adjusted life years in South Africa
Source: BMC Public Health. 2016 May 31;16:405. doi: 10.1186/s12889-016-3085-y (PMC4886444; doi:10.1186/s12889-016-3085-y)
Supplement: Additional file 1: — Estimates of the parameters used in the model.docx. Tables displaying parameter estimates used as inputs for the model. (DOCX 24 kb) [file 12889_2016_3085_MOESM1_ESM.docx]

Additional File 1 Estimates of the parameters used in the model

Table S1 Price elasticities and energy intake conversion factor used in the model

| Parameter | Mean Value(SD) |
| --- | --- |
| SSB own price elasticity | -1.30 (0.11) |
| Milk cross-price elasticity | 0.13 (0.1) |
| Fruit juice cross-price elasticity | 0.39 (0.19) |
| Diet drinks cross-price elasticity | -0.42 (0.10) |
| Daily energy intake required for 1 kg change in weight (kJ/kg/day) | 1. (2.96) |

**Table S2 Stroke relative risk, incidence, prevalence and case fatality rate baseline estimates**

| **Age** | **Relative risk per BMI-unit increase above 21(95% CI)**  **Male and female** | **Incidence Male** | **Incidence Female** | **Prevalence Male** | **Prevalence Female** | **Case fatality rate**  **Male** | **Case fatality rate**  **Female** |
| --- | --- | --- | --- | --- | --- | --- | --- |
| **0-4** | - | 0 | 0 | 0 | 0 | 0 | 0 |
| **5-9** | - | 0 | 0 | 0 | 0 | 0 | 0 |
| **10-14** | - | 0 | 0.0001 | 0 | 0.0002 | 0.0031 | 0.0019 |
| **15-19** | - | 0.0001 | 0.0003 | 0.0002 | 0.001 | 0.0247 | 0.026 |
| **20-24** | - | 0.0004 | 0.0007 | 0.0012 | 0.0026 | 0.0631 | 0.1211 |
| **25-29** | 1.13 (1.11-1.16) | 0.0007 | 0.0016 | 0.0028 | 0.0043 | 0.1191 | 0.2805 |
| **30-34** | 1.13 (1.10-1.15) | 0.0011 | 0.0029 | 0.0043 | 0.0065 | 0.1971 | 0.3711 |
| **35-39** | 1.12 (1.10-1.14) | 0.0017 | 0.003 | 0.0055 | 0.0085 | 0.2393 | 0.3123 |
| **40-44** | 1.11 (1.09-1.13) | 0.0032 | 0.0024 | 0.0089 | 0.01 | 0.2248 | 0.2061 |
| **45-49** | 1.10 (1.08-1.12) | 0.0049 | 0.0019 | 0.0164 | 0.0114 | 0.1973 | 0.1455 |
| **50-54** | 1.09 (1.08-1.11) | 0.0051 | 0.0026 | 0.0234 | 0.0136 | 0.1769 | 0.1261 |
| **55-59** | 1.08 (1.07-1.10) | 0.0037 | 0.004 | 0.0251 | 0.0203 | 0.154 | 0.1194 |
| **60-64** | 1.08 (1.06-1.09) | 0.0039 | 0.0037 | 0.0251 | 0.0252 | 0.1472 | 0.1303 |
| **65-69** | 1.07 (1.06-1.08) | 0.0041 | 0.005 | 0.0269 | 0.028 | 0.1346 | 0.1438 |
| **70-74** | 1.06 (1.05-1.07) | 0.004 | 0.0057 | 0.0284 | 0.0325 | 0.1365 | 0.1616 |
| **75-79** | 1.05 (1.04-1.06) | 0.0043 | 0.0057 | 0.0284 | 0.0322 | 0.1519 | 0.1854 |
| **80+** | 1.04 (1.03-1.05) | 0.0046 | 0.0061 | 0.0284 | 0.032 | 0.1605 | 0.1876 |

CI is confidence interval, BMI is body mass index.

**Table S3 All-cause pYLD, all-cause mortality rate and stroke-related healthcare cost estimates used in the model**

| **Age** | **pYLD Male** | **pYLD Female** | **All-cause mortality rate**  **Male** | **All-cause mortality rate**  **Female** | **Healthcare costs per person (ZAR)**  **Male and female** |
| --- | --- | --- | --- | --- | --- |
| **0** | - | - | 0.0427 | 0.0365 | - |
| **1-4/0-4** | 0.0671 | 0.0654 | 0.0044 | 0.0041 | R 13 184.74 |
| **5-9** | 0.0678 | 0.0587 | 0.0011 | 0.0010 | R 13 184.74 |
| **10-14** | 0.0778 | 0.0763 | 0.0011 | 0.0009 | R 13 184.74 |
| **15-19** | 0.0824 | 0.0893 | 0.0020 | 0.0018 | R 28 846.12 |
| **20-24** | 0.0962 | 0.1136 | 0.0043 | 0.0051 | R 28 846.12 |
| **25-29** | 0.1327 | 0.1430 | 0.0085 | 0.0104 | R 16 770.45 |
| **30-34** | 0.1520 | 0.1526 | 0.0142 | 0.0135 | R 16 770.45 |
| **35-39** | 0.1739 | 0.1573 | 0.0183 | 0.0140 | R 24 401.26 |
| **40-44** | 0.2026 | 0.1877 | 0.0200 | 0.0136 | R 24 401.26 |
| **45-49** | 0.2027 | 0.1924 | 0.0222 | 0.0135 | R 31 252.54 |
| **50-54** | 0.1966 | 0.1962 | 0.0255 | 0.0144 | R 31 252.54 |
| **55-59** | 0.2162 | 0.2090 | 0.0318 | 0.0176 | R 33 274.76 |
| **60-64** | 0.2170 | 0.1299 | 0.0387 | 0.0227 | R 33 274.76 |
| **65-69** | 0.2305 | 0.2272 | 0.0521 | 0.0324 | R 32 450.71 |
| **70-74** | 0.2194 | 0.2332 | 0.0611 | 0.0406 | R 32 450.71 |
| **75-79** | 0.2126 | 0.2446 | 0.0878 | 0.0668 | R 32 450.71 |
| **80-84** | 0.2043 | 0.2749 | 0.1310 | 0.0931 | R 32 450.71 |
| **85+** | - | - | 0.2781 | 0.1962 | R 32 450.71 |
|  |  |  |  |  |  |

pYLD is prevalent years lived with disability, T2DM is type 2 diabetes and ZAR is South African Rands
